# Diversity of fungal communities on Cabernet and Aglianico grapes from vineyards located in Southern Italy

**DOI:** 10.3389/fmicb.2024.1399968

**Published:** 2024-04-25

**Authors:** Massimo Iorizzo, Diletta Bagnoli, Franca Vergalito, Bruno Testa, Patrizio Tremonte, Mariantonietta Succi, Gianfranco Pannella, Francesco Letizia, Gianluca Albanese, Silvia Jane Lombardi, Raffaele Coppola

**Affiliations:** ^1^Department of Agricultural, Environmental and Food Sciences, University of Molise, Campobasso, Italy; ^2^Department of Science and Technology for Sustainable Development and One Health, University Campus Bio-Medico of Rome, Rome, Italy

**Keywords:** Aglianico, Cabernet, biogeography, fungi, grape microbiota, NGS, *terroir*, metataxonomic

## Abstract

Grape-associated microbial community is influenced by a combination of viticultural, climatic, pedological and anthropological factors, collectively known as *terroir*. Therefore, grapes of the same cultivar grown in different areas can be appreciated for their distinctive biogeographic characteristics. In our previous study, we showed that the phenotypic response of Aglianico and Cabernet grapevines from Molise and Sicily regions is significantly influenced by the prevailing pedoclimatic conditions, particularly soil physical properties. However, the scale at which microbial communities differ could be important in clarifying the concept of *terroir*, including whether it is linked to the grape variety present in a particular vineyard. To explore this further, in the research presented here, a comparative study on the fungal communities inhabiting the berry surfaces of Cabernet and Aglianico cultivars was conducted on different vineyards located in Southern Italy (Molise, Sicily and Campania regions, the first two of which had been involved in our previous study) by using high-throughput sequencing (HTS) and multivariate data analysis. The descriptive approach through relative abundance analysis showed the most abundant *phyla* (Ascomycota, Basidiomycota, and Chytridiomycota), families (*Cladosporiaceae*, *Saccotheciaceae*, *Pleosporaceae*, *Saccharomycodaceae*, *Sporidiobolaceae*, *Didymellaceae*, *Filobasidiaceae*, *Bulleribasidiaceae*, and *Saccharomycetaceae*) and genera (*Cladosporium*, *Aureobasidium*, *Alternaria*, *Stemphylium* and *Filobasidium*) detected on grape berries. The multivariate data analysis performed by using different packages (phyloseq, Vegan, mixOmics, microbiomeMarker and ggplot2) highlighted that the variable “vineyard location” significantly affect the fungal community, while the variable “grape variety” has no significant effect. Thus, some taxa are found to be part of specific vineyard ecosystems rather than specific grape varieties, giving additional information on the microbial contribution to wine quality, thanks to the presence of fermentative yeasts or, conversely, to the involvement in negative or detrimental roles, due to the presence of grape-deriving fungi implied in the spoilage of wine or in grapevine pathogenesis. In this connection, the main functions of core taxa fungi, whose role in the vineyard environment is still poorly understood, are also described.

## 1 Introduction

The grapevine (*Vitis vinifera*) phyllosphere is colonized by bacteria and fungi that substantially modulate grapevine health, fruit development and ripening, as well as the quality of grapes and wine (Barata et al., 2012; Martiniuk et al., 2023). The microbiome of a grapevine plant has direct and indirect relationships with its host and specific microbial biodiversity linked to a particular vineyard location is reported to be a crucial aspect, in conjunction with edaphic, climatic and human factors, in the concept of wine *terroir* (Gilbert et al., 2014). The concept of *terroir* is essential in viticulture since it links the sensory qualities of wine to the environmental conditions in which the grapes are grown (Van Leeuwen and Seguin, 2006).

Several scientific studies have shown that non-random biogeographic distribution patterns of microbial communities of grape surface in vineyards can be modulated by a combination of numerous factors, including geographical location, farming system, soil, cultivar, vintage and climate at various levels (Bokulich et al., 2014; Martins et al., 2014; Knight et al., 2015; Singh et al., 2018). These factors also affect the biology of the vines and the composition of the grapes, which in turn affect the microbiota, and, consequently, the qualitative properties of grapes and wine (Belda et al., 2017). This cycle has given rise to the concept of microbial-wine-terroir, intended as the close correlation between microbes, wine and territories (Gilbert et al., 2014; Knight et al., 2015).

To know the biogeographical distribution of vineyard microbial communities in specific regions, it is important to understand whether the vines themselves select different microorganisms according to their physiological responses, different environmental conditions and viticultural practices (Gilbert et al., 2014; Zarraonaindia et al., 2015; Pacifico et al., 2019; Vitulo et al., 2019). Therefore, in recent years, there has been a surge in the search for regional microbial signatures and microbial biogeography of wine and we now have a greater understanding of microbial diversity among wine-producing regions to begin to understand how this biodiversity can contribute to wine quality and regional characterization (Bokulich et al., 2014, 2016). Native yeasts, residing in a niche site, represent an important component of the microbiota of a vineyard, as they can influence the chemical and sensory profile and the so-called typicality of wine in a unique, reproducible, and identifiable way (Belda et al., 2017). Several studies have shown that the microbiota involved during the early fermentation stages, which is partially determined by endophytic plant-borne yeasts and bacteria, can comply with a well-delineated biogeography reflecting the signatures of different winegrowing regions with a minor influence from the grape variety and vintage year (Barata et al., 2012; Bokulich et al., 2014; Pretorius, 2020; Kamilari et al., 2021).

The diversity of the grape microbiota has long been studied using culture-dependent techniques (Barata et al., 2012; La Hens et al., 2014; Takahashi et al., 2014; Testa et al., 2014; Camilo et al., 2022), which have the disadvantage of being limited by the cultivability of microorganisms (Cocolin et al., 2013). Indeed, although these techniques are widely used for rapid and sensitive profiling of microbial communities, they are unable to detect a significant fraction of the microbial species present on grapes as rare taxa, low-abundance taxa and living but non-viable microorganisms (Bodor et al., 2020). The limitations of the conventional methods have led to the development of high-throughput sequencing (HTS)-based approaches to study the structure of microbial communities (Bleidorn, 2015). These innovative techniques allowed the scientific community to retrieve novel information about microbial communities in all different kinds of environments. As a result, the application of HTS methods for in-depth assessment of the grape and wine microbiome has increased in recent years (Salvetti et al., 2016; Morgan et al., 2017; Dissanayake et al., 2018; Kamilari et al., 2021). In a previous study we already assessed the impact of pedoclimatic conditions on the enological performance of two red cultivars grown throughout Southern Italy (Iorizzo et al., 2023). In this study, we obtained new information by adding other wine-grape samples of two cultivars (Cabernet and Aglianico) to determine relationships between spatial proximity and fungal composition through amplicons sequencing of the internal transcribed spacer (ITS2) region using a HTS approach combined with bioinformatics.

## 2 Materials and methods

### 2.1 Berry sample collection and processing

Grape berries from cultivars Aglianico and Cabernet were collected in two different vineyards of Southern Italy (Sicily and Molise, 37° 40′; 400–700 m a.s.l. and 41° 42′; 606 m a.s.l., respectively) at the right maturation time, following the BBCH scheme described by Lorenz et al. (1995) (BBCH stage 89, berries ripe for harvest), during the 2020 growing season. Grape barriers from a third farm located in the Campania region (41° 13′; 100 m a.s.l.) and devoted to the production of Aglianico and Cabernet were also withdrawn in the same period. For each farm and for each cultivar, 30 clusters were harvested from different positions of the vineyard and from random positions on the plant to ensure the representation of the entire vineyard as previously described (Iorizzo et al., 2023). Briefly, at least ten berries were randomly selected from different parts of the cluster, avoiding those with visible damage and/or signs of pathogen infection, and pooled with berries from the other plants. For subsequent analyses, two different samples of 50 whole berries were taken and immediately transported to the laboratory, frozen in liquid nitrogen, and stored at −80°C for subsequent analyses.

### 2.2 Metataxonomic and bioinformatic analyses of grape fungal communities

Fungal community composition was analyzed by a culture-independent approach using next generation sequencing (NGS), as described in a previous study (Iorizzo et al., 2023). Total genomic DNA was extracted using the Stool DNA Isolation Kit (Norgen, Biotek Corp., Thorold, ON, Canada) according to the manufacturer’s instructions. Two replicates were produced for each sample. DNA was extracted from 200 mg of the homogenized sample. Extracted DNA was verified by agarose gel electrophoresis. The purity and amount of DNA was measured using a NanoDrop (NanoDrop 2000/2000c, Thermo Fisher Scientific, Italy) and standardized to a concentration of 10 ng/μl. The ITS2 region of the rRNA was amplified using primers 2024F and 2409R (White et al., 1990; Zhang et al., 2018). Amplicons were sequenced using the Illumina MiSeq PE300 platform (Illumina, San Diego, CA, USA) at Biodiversa s.r.l. (Rovereto, Italy). Raw paired-end reads obtained from NGS were demultiplexed using the QIIME 2 (ver. 2022.2) pipeline (Caporaso et al., 2010) and then were analyzed to evaluate the quality of sequencing with the DADA2 plugin (*qiime dada2 denoise-paired*) in QIIME2 (Callahan et al., 2016). For the quality control of reads, the Quality Score value of 25 was used as a threshold and the low-quality regions of sequences were removed. Reads were truncated at position 300 and 254 for the forward and reverse read, respectively. Moreover, to remove the base that belongs to the primer sequences, the first 5 nucleotides of reads were removed. At the end of DADA2 analysis, an amplicon sequence variant (ASV) table was obtained which recorded the frequencies of any ASV observed in each sample. The ASV table was filtered with the plugin qiime *feature-table filter-features* to discard features with frequency lower than 10 (0.001%).

ASVs were classified using the QIIME2 naïve Bayes classifier (*qiime feature-classifier fit-classifier-naive-bayes*), trained on the UNITE database (ver. 9.0, release 16-10-2022) (Abarenkov et al., 2022).

The new raw data of Campania samples were deposited in Mendeley Data with the accession number doi:10.17632/6jcyrwj2gh.

### 2.3 Statistical analysis

Statistical analysis and graph processing were conducted on R (Version 4.2.3) using RStudio software (Version 2022.07.0). Several packages were used in the analysis: phyloseq (Version 1.42.0) to facilitate the import, storage, handling and analysis of the microbiome data (McMurdie and Holmes, 2013), as well as to determine alpha diversity. For the latter, both Chao1 and Shannon indexes were calculated. Vegan (Version 2.6-4) package was used for the permutational multivariate analysis of variance (PERMANOVA) using the Bray Curtis metric distance. The mixOmics package was used for sparse partial least squares discriminant analysis (Rohart et al., 2017) and the microbiomeMarker (Version 1.4) package for the Linear Discriminant Analysis (LDA) effect size (SE). The ggplot2 (Version 3.4.4) package was used for graphical representation of the data.

## 3 Results

### 3.1 Bioinformatic analysis

In the year 2020 two biological replicate samples of grapes were collected from two vineyards (Aglianico and Cabernet) located in Southern Italy (Sicily, Campania and Molise regions, Supplementary Table 1). Surface fungi community composition of grapes was analyzed through high-throughput amplicon sequencing of the ITS2 region.

A total of 923,559 paired-end sequences were obtained from sample sequencing. The average of reads per sample was 76,963, ranging from 54,059 to 123,628. After reads quality check, denoising and chimera filtering (Supplementary Figure 1), 465,805 paired-end sequences were obtained (Supplementary Table 2) corresponding to 419 ASVs. The ASVs were filtered with the aim to discard features with frequency lower than 10. At the end of bioinformatic analysis, a feature table of 465,469 reads, corresponding to 323 ASVs, was obtained and used for the subsequent analysis. The rarefaction curves reached the plateau for all assayed samples, suggesting that the sequencing depth was appropriate (Supplementary Figure 2).

### 3.2 Grape fungi community profile

**FIGURE 1:**
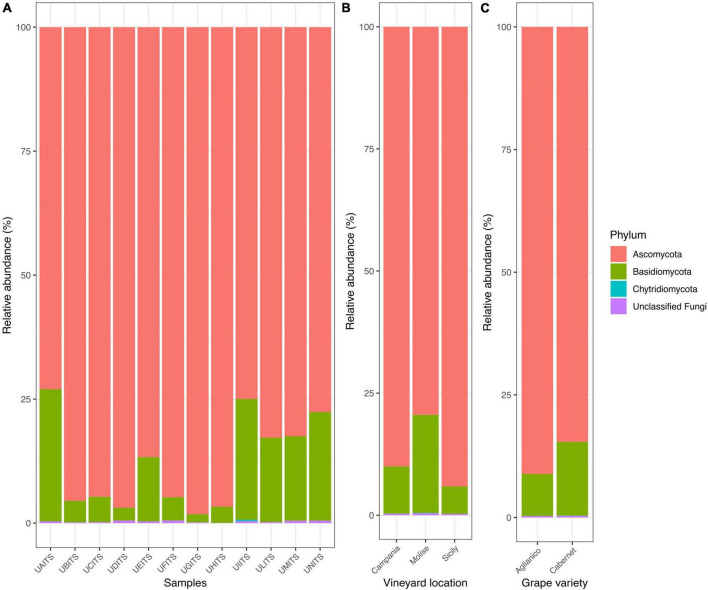
Stacked bar plot illustrates the distribution of the relative abundance of the grape fungi community at the *phylum* level. The relative abundance is reported for panel **(A)** single samples, **(B)** samples grouped by vineyard location (VL), and **(C)** samples grouped by grape variety (GV). The sample codes are specified as follow: UAITS, Cabernet_Campania_1; UBITS, Cabernet_Campania_2; UCITS, Aglianico_Campania_2; UDITS, Aglianico_Campania_3; UEITS, Cabernet_Sicily_1; UFITS, Cabernet_Sicily_3; UGITS, Aglianico_Sicily_1; UHITS, Aglianico_Sicily_2; UIITS, Cabernet_Molise_2; ULITS, Cabernet_Molise_3; UMITS, Aglianico_Molise_1; UNITS, Aglianico_Molise_2. See Supplementary Table 1 for more details.

From the taxonomy classification 3 *phyla*, 17 classes, 45 orders, 96 families, 122 genera and 172 species were identified. In general, the analysis of fungal community confirmed our previous findings (Iorizzo et al., 2023). In fact, Ascomycota remained the most abundant *phylum*, representing 87.9% ± 9.4% of total reads, followed by Basidiomycota 11.8% ± 9.3% and Chytridiomycota 0.02% ± 0.08% in all analyzed samples. The remaining 0.3% ± 0.2% of the reads were represented by unclassified fungi (Figure 1A and Supplementary Table 3).

The new data processed in this study considered two variables: vineyard location (VL) and grape variety (GV). Regarding the results of relative abundance with respect to the variable VL (Figure 1B and Supplementary Table 4), we found that the grape samples from Campanian vineyard were characterized by 90% of reads belonging to the Ascomycota and about 9.7% of reads were referable to the Basidiomycota. Similarly, in the grape samples from Sicilian vineyard, an average of 94% of the reads were associated with the *phylum* Ascomycota and only 5.6% of them belonged to the Basidiomycota. A slightly different result was found in the Molisian samples as Ascomycota accounted for around 74% of the reads, while around 20% of the reads belonged to Basidiomycota. When the samples were grouped according to the GV (Figure 1C and Supplementary Table 5), we found that Ascomycota represented 91.1 and 84.6% of the *phyla* for the Aglianico and Cabernet cultivars, respectively. Basidiomycota, on the other hand, were around 8.6% in the Aglianico samples and 15% in the Cabernet samples.

**FIGURE 2:**
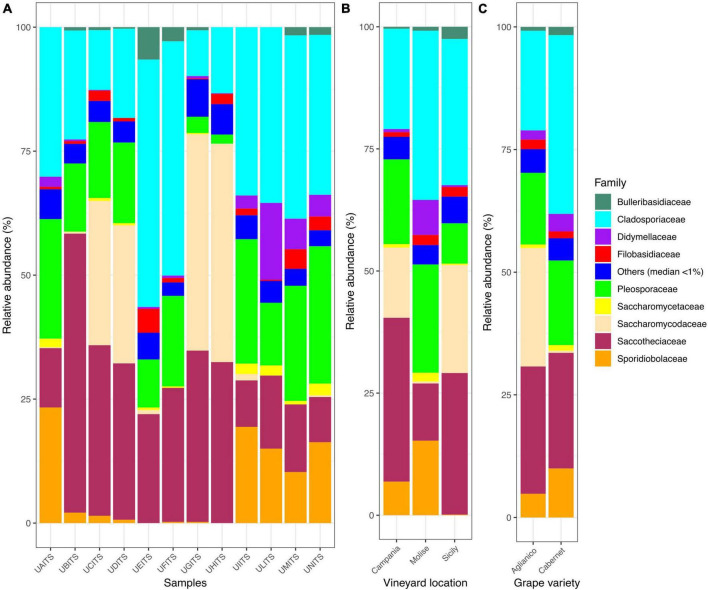
Stacked bar plot illustrates the distribution of the relative abundance of the grape fungi community at the family level. The group indicated as “Others (median < 1%)” encompasses families that show a relative abundance below median value 1. The relative abundance is reported for panel **(A)** single samples, **(B)** samples grouped by vineyard location (VL), and **(C)** samples grouped by grape variety (GV). The sample codes are specified as follow: UAITS, Cabernet_Campania_1; UBITS, Cabernet_Campania_2; UCITS, Aglianico_Campania_2; UDITS, Aglianico_Campania_3; UEITS, Cabernet_Sicily_1; UFITS, Cabernet_Sicily_3; UGITS, Aglianico_Sicily_1; UHITS, Aglianico_Sicily_2; UIITS, Cabernet_Molise_2; ULITS, Cabernet_Molise_3; UMITS, Aglianico_Molise_1; UNITS, Aglianico_Molise_2. See Supplementary Table 1 for more details.

Regarding the classification at the family level, out of 96 families identified, only 9 of them enclosed about 95% of total reads. In detail, *Cladosporiaceae* was the most abundant family, representing an average of 28.4% ± 13.5 of the total reads, followed by *Saccotheciaceae* 24.8% ± 14.0%, *Pleosporaceae* 15.9% ± 8.3%, *Saccharomycodaceae* 12.3% ± 18.2%, *Sporidiobolaceae* 7.40% ± 8.80%, *Didymellaceae* 2.7% ± 4.5%, *Filobasidiaceae* 1.70% ± 1.53%, *Bulleribasidiaceae* 1.2% ± 1.9%, and *Saccharomycetaceae* 0.9% ± 0.9% (Figure 2A and Supplementary Table 6). With regards to the distribution of the 9 most abundant families according to the VL (Figure 2B and Supplementary Table 7), we observed that *Cladosporiaceae* were present with a prevalence of about 20.6, 34.7, and 29.9% in vineyards of Campania, Molise and Sicily regions, respectively. *Saccotheciaceae* showed a prevalence of 33.6% in samples from Campanian vineyard, 11.7% in Molisian vineyard and 29% in Sicilian vineyard. Reads associated with *Pleosporaceae* were present at levels of 17.4, 22.1, and 8.3% in samples from vineyards located in Campania, Molise and Sicily regions, respectively. The results regarding the distribution of *Saccharomycodaceae* among the samples were particularly interesting. In fact, a consistent presence of reads attributable to *Saccharomycodaceae* were detected only in Campanian (14.4%) and Sicilian (22.1%) vineyards. On the contrary, only 0.4% of *Saccharomycodaceae* were observed in Molisian vineyard. *Sporidiobolaceae* were detected at relevant presence in Campanian (6.9% ± 10.9%) and Molisian (15.2% ± 3.4%) vineyards, while their presence on grapes from Sicilian farm was only 0.1%. Another interesting result concerns the *Didymellaceae* family. In this regard, a consistent presence of reads was observed only in Molisian vineyard (7.2%), while a poor relative abundance was detected in the other samples (0.6% Campania and 0.4% Sicily).

Investigating the distribution of the nine families in relation to the variable VL (Figure 2C and Supplementary Table 8), we observed that *Saccharomycodaceae* were particularly abundant (24.1%) in Aglianico grapevine and almost undetectable in Cabernet samples (0.4%).

**FIGURE 3:**
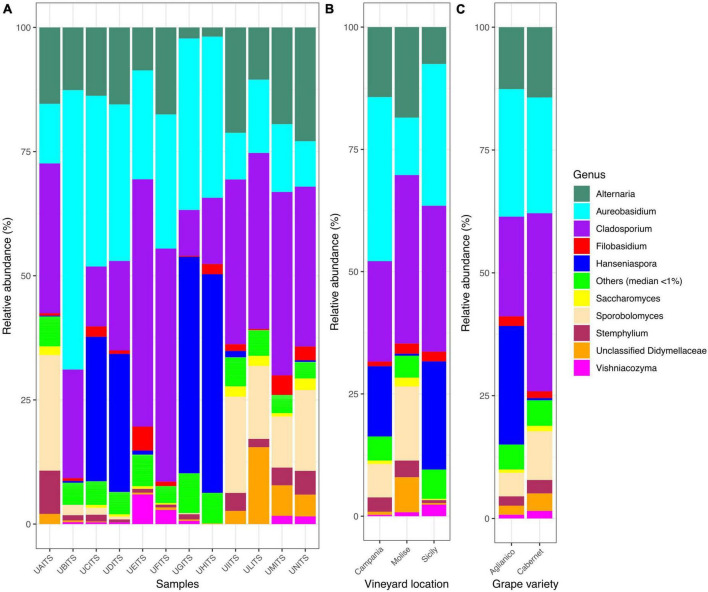
Stacked bar plot illustrates the distribution of the relative abundance of the grape fungi community at the genus level. The group indicated as “Others (median < 1%)” encompasses genera that show a relative abundance below median value 1. The relative abundance is reported for panel **(A)** single samples, **(B)** samples grouped by vineyard location (VL), and **(C)** samples grouped by grape variety (GV). The sample codes are specified as follow: UAITS, Cabernet_Campania_1; UBITS, Cabernet_Campania_2; UCITS, Aglianico_Campania_2; UDITS, Aglianico_Campania_3; UEITS, Cabernet_Sicily_1; UFITS, Cabernet_Sicily_3; UGITS, Aglianico_Sicily_1; UHITS, Aglianico_Sicily_2; UIITS, Cabernet_Molise_2; ULITS, Cabernet_Molise_3; UMITS, Aglianico_Molise_1; UNITS, Aglianico_Molise_2. See Supplementary Table 1 for more details.

In relation to genera classification, out of 122 genera detected, only 10 groups were present in almost all samples (Figure 3A and Supplementary Table 9). In detail, *Cladosporium* was present with an average of 28.3%, followed by *Aureobasidium* (24.8%), *Alternaria* (13.5%), *Hanseniaspora* (12.3%), *Sporobolomyces* (7.3%), unclassified *Didymellaceae* (2.7%), *Stemphylium* (2.3%) *Filobasidium* (1.7%), *Vishniacozyma* (1.1%) and *Saccharomyces* (0.9%). Genera grouped as “Others (median < 1)” showed about 5% of average relative abundance. Grouping the genera distribution according to the VL variable (Figure 3B and Supplementary Table 10), it was observed that in Campanian vineyard the genus *Aureobasidium* was the most abundant (33.5%), followed by *Cladosporium* (20.5%), *Hanseniaspora* (14.4%), and *Alternaria* (14.3%). Samples from Molise vineyard were also characterized by the high presence of *Cladosporium* (34.4%), *Alternaria* (18.5%) and *Aureobasidium* (11.7) genera, but contrary to the Campania samples, a high amount of *Sporobolomyces* (15.2%) and a low presence of *Hanseniaspora* (0.4%) was observed. With regard to the samples from Sicilian vineyard, *Cladosporium* (29.8%), *Aureobasidium* (29.0%) and *Hanseniaspora* (22.1%) were the most abundant genera, instead *Alternaria* (7.5%) and *Sporobolomyces* (0.1%) were present in a small number. When the dataset was stratified according to the GV variable (Figure 3C and Supplementary Table 11), *Cladosporium* was the most abundant genus (34.3%) in the Cabernet grape variety, followed by *Aureobasidium* (18.3%) and *Alternaria* (14.6%), while in the Aglianico samples, a significant amount of reads attributable to the genus *Hanseniaspora* (28.4%) was recorded in addition to a high presence of *Aureobasidium* (32.0%), *Cladosporium* (15.7%) and *Alternaria* (14.6%).

### 3.3 Alpha diversity of grapevine according to the vineyard location and grape variety

**FIGURE 4:**
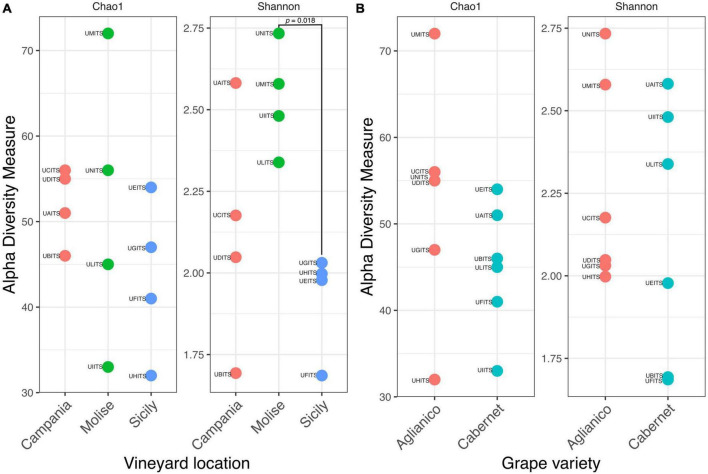
Alpha diversity of grapes calculated according to the variables **(A)** vineyard location (VL) and **(B)** grape variety (GV). Species richness and diversity were calculated with Chao1 and Shannon indexes, respectively. The sample codes are specified as follow: UAITS, Cabernet_Campania_1; UBITS, Cabernet_Campania_2; UCITS, Aglianico_Campania_2; UDITS, Aglianico_Campania_3; UEITS, Cabernet_Sicily_1; UFITS, Cabernet_Sicily_3; UGITS, Aglianico_Sicily_1; UHITS, Aglianico_Sicily_2; UIITS, Cabernet_Molise_2; ULITS, Cabernet_Molise_3; UMITS, Aglianico_Molise_1; UNITS, Aglianico_Molise_2. See Supplementary Table 1 for more details.

The estimation of intragroup diversity (alpha diversity) was carried out to verify the hypothesis that the fungi richness and biodiversity vary within the VL (Campania, Molise, and Sicily) and the GV (Aglianico and Cabernet). For this purpose, Chao1 index was used to estimate the species richness and the Shannon index was used to estimate the diversity. Results are reported in Figure 4A and show that there were no significant differences (Anova test, *p* = 0.516) in richness (Chao1) between GV in the three vineyards. In contrast, a significant difference (Anova test, *p* = 0.021) of diversity (Shannon) between VL was found. Specifically, the *post hoc* test showed that the Molisian vineyard was characterized by a high diversity (TukeyHSD test, *p* = 0.018) compared to the Sicilian one. No significant differences (TukeyHSD test, *p* > 0.05) were found between vineyards from Molise and Campania regions or between Campania and Sicily regions. Regarding the alpha diversity calculated from the two GV, results showed that there were no significant differences (*t*-test, *p* > 0.05) for species richness and diversity (Figure 4B).

### 3.4 Beta diversity

**FIGURE 5:**
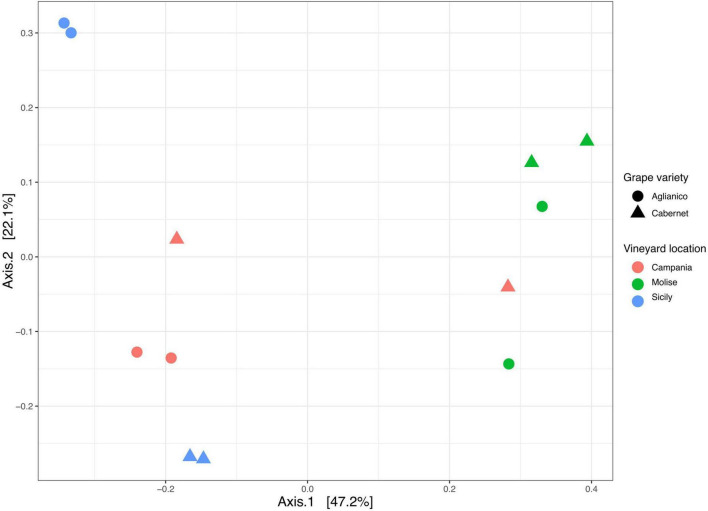
Principal Coordinate Analysis (PCoA) based on Bray Curtis distance generated with ASVs (at 99% identity) present in samples grouped according to vineyard location (VL) and grape variety (GV).

Beta diversity based on Bray Curtis distance was calculated to evaluate the degree of variation in species composition among the samples. Results showed that the fungal population segregated mainly according to the VL and slightly according to the GV (Figure 5). In detail, the PCoA plot shows a good separation of samples along axis 1 (explaining 47% of variation), while a less pronounced separation was observed along axis 2 (explaining 22% of variation). The effect and the significance of the variable VL (Campanian, Molisian and Sicilian vineyards) and GV (Aglianico and Cabernet) on the fungi was investigated through the Permutational Multivariate Analysis of Variance (PERMANOVA). The results revealed that the variable VL significantly (*p* = 0.0001) affected fungal communities, while the variable GV had no significant effect (*p* > 0.05). However, the interaction VL: GV showed a significant (*p* = 0.001) effect on the overall variance of the dataset (Supplementary Table 12). From multiple comparison tests, it was highlighted that samples from Molise vineyard were significantly different (*p* = 0.039) from those of Campanian and Sicilian vineyards. No differences (*p* > 0.05) were found between Campania and Sicily vineyards.

### 3.5 Discriminant analysis

**FIGURE 6:**
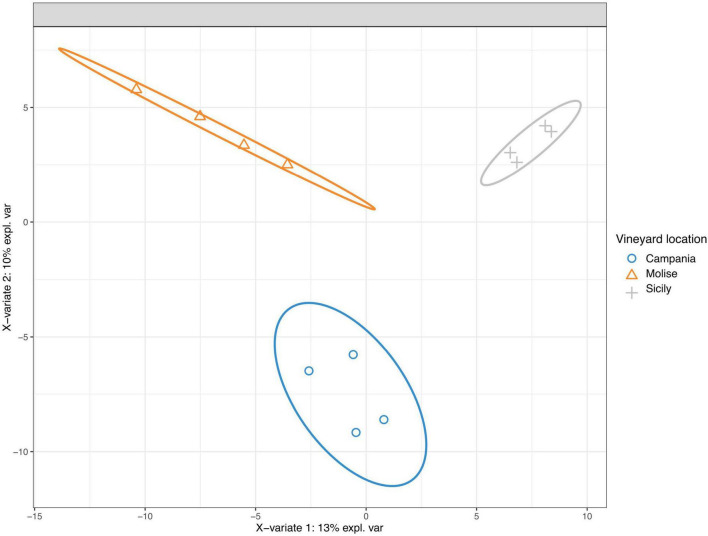
Sparse partial least-squares discriminant analysis (sPLS-DA) of read counts transformed using cumulative sum scaling normalization at the sequence variant level. The ellipse shows the 95% confidence level.

The taxonomic groups driving differences between fungal communities were investigated with the sparse Partial Least Squares Discriminant Analysis (sPLS-DA). The results show that the variable VL influences the entire community (Figure 6). Grape samples from Molise vineyard separated from those of Sicilian vineyard along component 1 (X-variate 1), while grape samples from Campania vineyard separated from those of the other vineyards along component 2 (X-variate 2).

**FIGURE 7:**
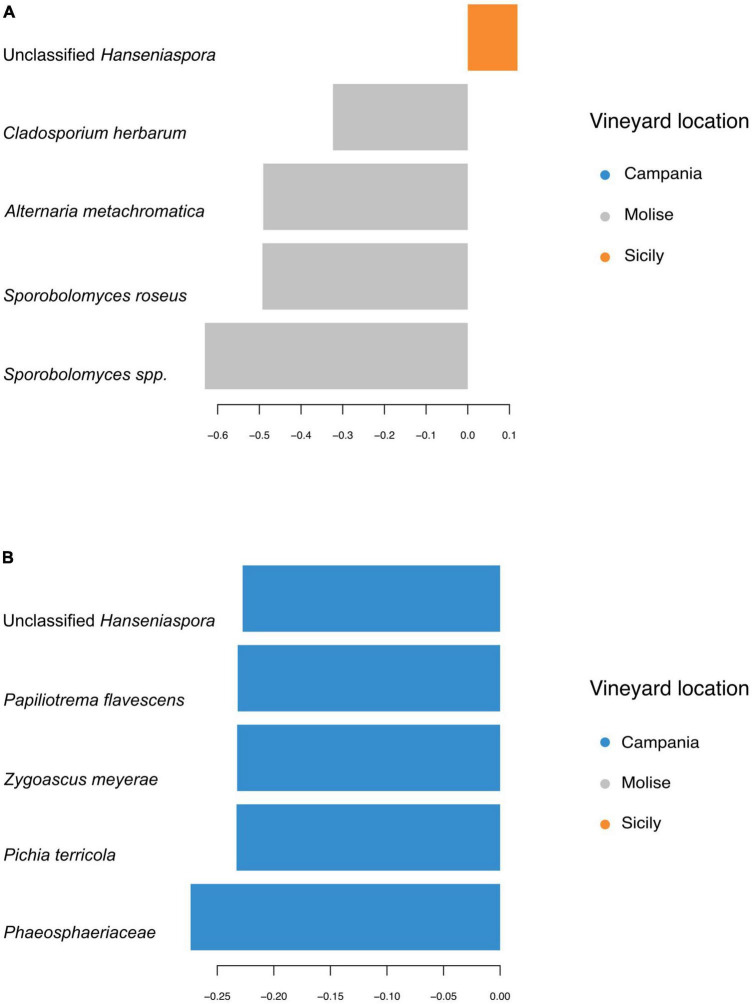
First five sequence variants contributing to the separation along with panel **(A)** component 1, and **(B)** component 2 of sPLS-DA from Figure 6. Bar length indicates loading coefficient ranked by importance, bottom to top; bar color indicates the group in which the sequence variant has the highest median abundance.

Taxa contributions on the first 2 components are reported in Figures 7A, B. Fungi that mainly increased in abundance in samples from Molise vineyard included *Sporobolomyces roseus*, *Alternaria metacromatica*, and *Cladosporium herbarum*, whilst fungi associated to samples from Sicilian vineyard belonged to the genus *Hanseniaspora*. Regarding taxa associated to Campanian vineyard, sPLS-DA analysis reported *Phaeosphaeriaceae* family and *Pichia terricola, Zygoascus meyerae, Papiliotrema flavescens* and *Hanseniaspora* spp. as the top 5 most abundant groups.

**FIGURE 8:**
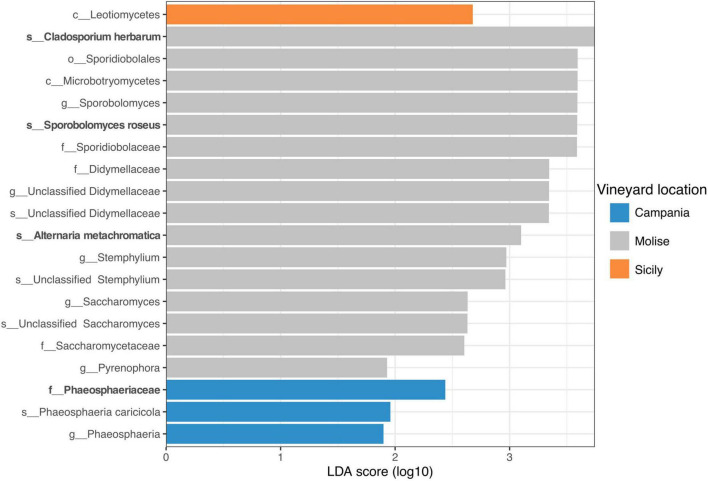
Linear discriminant analysis (LDA) effect size (LEfSe). Horizontal bars represent the effect size for each taxon. The cut off on the logarithmic LDA score for discriminative features was set to 1.5. The change of taxon relative abundance was statistically significant at *p* < 0.05. The name of the taxon level is abbreviated as p__phylum; c__class; o__order; f__family, and g__genus. Taxa name in bold were confirmed by sPLS-DA analysis reported in Figure 7.

Overall, the results obtained from sPLS-DA were supported by the Linear discriminant analysis (LDA) effect size (LEfSe). LEfSe results revealed that 20 fungal taxa were mainly present in the samples of the three vineyards under study with significant differences (*p* < 0.05) at a threshold value of 1.5. In detail, one fungal taxon was associated with the Sicilian vineyard, three fungal taxa with the Campanian vineyard and 16 fungal taxa were significantly associated with the Molisian vineyard (Figure 8). In particular, according to results obtained by sPLS-DA analysis, the *Phaeosphaeriaceae* family was associated with the vineyard located in Campania region, while *Phaeosphaeria caricicola*, that resulted also associated with Campanian samples though LEfSe analysis, was not confirmed with sPLS-DA analysis. Regarding the samples belonging to the Molise vineyard, both LEfSe and sPLS-DA analysis confirmed *Cladosporium herbarum, Sporobolomyces roseus*, and *Alternaria metachromatica* significantly correlated to VL. Instead, *Didymellaceae* family, *Sthemphylium* genus, *Saccharomycetaceae* family and *Pyrenophora* genus resulted correlated to the Molise vineyard only when LEfSe analysis was applied. With regard to samples belonging to the Sicilian vineyard, LEfSe analysis reported that only Leotiomycetes was associated with the VL, contrarily to what it was obtained from the sPLS-DA analysis.

## 4 Discussion

Microorganisms have a key role in the winemaking process as they drive fermentation and are able to influence the aroma and quality of wine (Verginer et al., 2010; Belda et al., 2017; Sherman et al., 2020). Grape berries are one of the main sources of microbial communities that affect the winemaking process, therefore the study of the microbial diversity of wine grapes is of particular interest (Morrison-Whittle and Goddard, 2018). The structure of microbial communities could be, in turn, influenced by several factors such as geographical location, climatic conditions, grape variety, viticultural practices (e.g., fungicides, herbicides, fertilizers) and many others (Bokulich et al., 2014; Pinto et al., 2014; Setati et al., 2015; Grangeteau et al., 2017; Vitulo et al., 2019). As far as wine is concerned, individual vineyards are the smallest scale through which wine diversity can be studied (Knight et al., 2020) and understanding the scale at which microbial communities differ could be important in clarifying the concept of *terroir*.

Due to the complexity of the microbial population found on grape berries, it is evident that traditional culture-dependent methods have limitations in studying microbial diversity. On the contrary, high-throughput sequencing (HTS), coupled with multivariate data analysis, represents a valid method for studying microorganisms in a variety of contexts. In the present study, we used both HTS and multivariate data analysis to investigate the fungi community structure of grape samples collected in three different vineyards located in Campania, Molise and Sicily regions (Southern Italy) and belonging to two red grape varieties (Aglianico and Cabernet). In general, the descriptive approach through relative abundance analysis confirmed what we already found in our previous study (Iorizzo et al., 2023), but in the present research, new information is derived from the addition of new samples from the Campanian vineyard. Moreover, the statistical approach used furnished new important information on the structure of fungal communities as related to vineyard location and grape variety.

In detail, we found that the main *phyla* identified on grape berries were represented by Ascomycota, Basidiomycota and Chytridiomycota. In particular, the most abundant *phylum* in our samples was Ascomycota (∼ 88%), followed by Basidiomycota (∼ 12%). Similarly, Gao et al. (2019) reported that Ascomycota, Basidiomycota and Zygomycota were the main *phyla* found on the surface of grape berries from six wine regions in Xinjiang - China. In addition, other authors have also found Ascomycota and Basidiomycota as the dominant fungal *phyla* on grape berries (Morrison-Whittle et al., 2017). Recently, Xu et al. (2022) conducted a study in which they reported that Ascomycota and Basidiomycota were the dominant fungi present on wine-grape surfaces.

Regarding the fungal community structure at the family level, we showed that *Cladosporiaceae*, *Saccotheciaceae*, *Pleosporaceae*, *Saccharomycodaceae* and *Didymellaceae* were the most representative families within the *phylum* Ascomycota, while *Sporidiobolaceae*, *Filobasidiaceae* and *Bulleribasidiaceae* were the most abundant ones among the *phylum* Basidiomycota. Similarly, in previous studies, other authors reported that *Pleosporaceae* and several members of *Saccotheciaceae* and *Cladosporiaceae* were the dominant fungi in Riesling grape berries (Kecskeméti et al., 2016). Interestingly, stratifying the dataset according to the variable VL, we observed that *Cladosporiaceae*, *Saccotheciaceae*, *Pleosporaceae*, and *Filobasidiaceae* were present in all samples (*n* = 12), suggesting that these 4 families represent the core taxa. Similar results were also obtained by Gao et al. (2019) in their work regarding the study of the fungal community structure of grape surfaces. In addition, Wei et al. (2022) reported that the core taxa of Cabernet Sauvignon grapes are retained at the genus level, although the fungi diversity changes during the berry development.

Studying the fungal community structure of grape berries at genus level, on a dataset stratified in accordance with the variable VL, we found that the main genera (about 71% of OTUs) were represented by *Cladosporium*, *Aureobasidium*, *Alternaria*, *Stemphylium*, and *Filobasidium*. Similarly, when the fungal community structure of grape berries was studied on a dataset stratified by the variable GV, it was observed that, apart from the genus *Stemphylium*, all other genera were conserved, indicating that *Cladosporium*, *Aureobasidium*, *Alternaria*, and *Filobasidium* constitute the core taxon. Some of these genera were also reported as the most common fungal organisms isolated from grapes in other parts of the world. For example, in a study conducted in different regions of Portugal, the authors reported that *Cladosporium, Alternaria*, and *Aureobasidium* were the main genera of Alvarinho grapes (Fernandes et al., 2023). In another recent work conducted in the Xinjiang region of China, the authors found that the dominant fungi on nine grape skins variety were represented by *Alternaria*, *Filobasidium*, *Aureobasidium*, *Erysiphe* and *Naganishia* (Xu et al., 2022). In Italy, Perpetuini et al. (2022) reported that the common genera of fungi detected in grape samples of Montepulciano d’Abruzzo cultivar (Abruzzo Region, Central Italy) were represented by *Hanseniaspora, Aureobasidium, Botrytis, Pichia, Cladosporium* and *Alternaria.* In another work conducted by Perpetuini et al. (2023), it was found that *Aureobasidium, Metschnikowia, Hanseniaspora, Botrytis, Cladosporium, Pichia, Alternaria, Epicoccum, Vishniacozyma* and *Candida* were the main genera detected on the minor grapevine cultivar cv. Nero Antico from Chieti (Abruzzo Region, Central Italy). Similarly, Rossetti et al. (2023) studied the fungal community associated with conventional and organic Trebbiano Abruzzese grapes from vineyards of Chieti (Abruzzo Region, Central Italy), and a core taxon of 8 genera was detected (*Zygosaccharomyces* spp., *Cladosporium* spp., *Botrytis* spp., *Hanseniaspora* spp., *Pichia* spp., *Alternaria* spp., *Candida* spp., *Aureobasidium* spp.) in all samples investigated.

For a better understanding of the fungal community structure of grape samples and to assess the hypothesis that the fungal population of grape berries differs between samples depending on vineyard location(VL) and/or grape variety (GV), we used several statistical tools based on both univariate and multivariate approaches.

In detail, the alpha diversity values indicated that there were no significant differences (*p* > 0.05) in terms of richness (Chao1) between the analyzed samples, highlighting that the number of species present in the samples was essentially the same, regardless the VL and the GV (Figure 4). On the other hand, alpha diversity as measured with the Shannon index, evidenced that the VL may have a significant effect on the fungal community composition, as grapes from Molisian vineyard have a greater diversity than grapes from Sicilian one (Figure 4). This result indicates that there is a greater presence of rare species in grape samples from Molisian vineyard than those observed in Sicilian samples.

The effect of VL on the diversity of fungal communities among samples was also supported by the beta diversity measurement. In fact, both PCoA and sPLS-DA analyses showed that samples are mainly grouped according to the VL (Figure 5). These results are consistent with those reported in other studies where it was demonstrated that the diversity of the microbial community is influenced by geographical location of vineyards (Bokulich et al., 2014; Mezzasalma et al., 2017; Kioroglou et al., 2019; Papadopoulou et al., 2022; Kazou et al., 2023). Similar results were also obtained by other authors who have reported that VL has a greater impact on the fungal community than the state of ripeness (Kioroglou et al., 2019).

In the present study, a strong association between some families genera or species and their VL was confirmed by the results obtained using both sPLS-DA and LEfSe. For instance, both sPLS-DA and LEfSe analyses evidenced that *C. herbarum*, *S. roseus* and *A. methacromatica* were associated to Molisian vineyard. Similarly, the Campanian vineyard showed a strong association with the *Phaeosphaeriaceae* family, while they were weakly associated with *Pichia terricola*, *Zygoascus meyerae* and *Papiliotrema flavescens*. Regarding the Sicilian vineyard, a weak association with *Hanseniaspora* genus or with Leotiomycetes was observed since the results were not confirmed by both analyses (sPLS-DA and LEfSe).

Apart from the well-known role of *Hanseniaspora* spp. on the quality of some wines (Lombardi et al., 2018; Testa et al., 2020), the exact functions of core taxa fungi or potential biomarker fungi in wine and winemaking are unknown or poorly investigated for most of the taxa identified in the present work, while some more information, even if sometimes in contrast, is available regarding their role on grapevines. For example, some studies reported that *Filobasidium* spp. was detected on undamaged Tempranillo grapes, but it was not involved in spontaneous must fermentation (Bougreau et al., 2019). Similarly, *Aureobasidium* would appear to have no impact on the fermentation process and the wine quality (Barata et al., 2012; Setati et al., 2015; Grangeteau et al., 2017). Also, *Alternaria* spp. is generally not associated with neither fermentation or spoilage of wine (Barata et al., 2012; Setati et al., 2015; Grangeteau et al., 2017), while *Cladosporium* genus is considered a spoilage of the winery environment (Barata et al., 2012; Grangeteau et al., 2017). Both *Cladosporium* spp. and *Alternaria* spp. are endophytic fungi detected in plants showing symptoms of grapevine trunk diseases (GTDs) although their direct role in the disease was not confirmed (Patanita et al., 2022). In other studies, these genera were detected on grapevines, but no visible symptoms of plant diseases were observed (Varanda et al., 2016). Some authors reported that the presence of *Cladosporium* genus, in particular *C. herbarum*, on grape berries, is associated with the *Cladosporium* rot (Barata et al., 2012). In our study, despite the association of *C. herbarum* with grape samples from Molise vineyard, no disease symptoms were visible on the grapes or grape plants. This could be attributed to the fact that the induction of disease may depend on many factors ascribable to the concurrent presence of this species with other microorganisms or with specific plant factors (Liu and Howell, 2021). Similar contrasting results were also reported regarding *Alternaria* spp., as some authors reported the potential role of *Alternaria* spp. as biological control agent against GTDs-associated fungi and other grapevine pathogens (Musetti et al., 2006; Bruez et al., 2014).

*Sporobolomyces* spp. have been detected in grape musts as well as at the beginning of wine fermentation (Li et al., 2018). These yeasts are not involved in the alcoholic fermentation, but they could contribute to the production of some metabolites that affect the aroma of wines. For instance, it is known that *Sporobolomyces roseus* can produce volatile compounds such as higher alcohols, acetate esters and thiols (Verginer et al., 2010). Moreover, there are evidence of its potential role as post-harvest biocontrol agent against *Penicillium expansum* (Sanzani et al., 2021).

## 5 Conclusion

Microbial *terroir* involves multiple interacting aspects, such as soil and plant-associated microbes and plant-microbe interactions. The present metataxonomic study explored the distribution of fungal communities at vineyard scale considering different vineyards located in Southern Italian regions dedicated to the production of Aglianico and Cabernet wines. Our results, although worthy of further investigation, highlighted the relevance of the vineyard location in the definition of grape-associated fungal communities. Moreover, from the results obtained in the present study, it appeared that the fungal community present on grape berries is more associated with vineyard location than with the grapevine cultivar, allowing to consider some fungal groups as territorial biomarkers regardless of the cultivar analyzed. For instance, in our study, the *Phaeosphaeriaceae* family was associated to the Campanian vineyard, while *Cladosporium herbarum, Sporobolomyces roseus*, and *Alternaria metachromatica* resulted significantly correlated to samples from Molisian vineyard and Leotiomycetes to the Sicilian one, and this for both varieties analyzed. Of course, further studies are needed, as numerous variables such as growing season, soil properties, plant status and other factors may contribute to the definition of fungal communities typical of vineyards located in near geographical areas.

## Data availability statement

The datasets presented in this study can be found in online repositories. The names of the repository/repositories and accession number(s) can be found below: https://data.mendeley.com/datasets/tp2rrdz8wp/2, doi: 10.17632/tp2rrdz8wp. https://data.mendeley.com/drafts/6jcyrwj2gh, doi: 10.17632/6jcyrwj2gh.

## Author contributions

MI: Conceptualization, Validation, Writing – original draft, Writing – review and editing. DB: Formal analysis, Writing – review and editing. FV: Formal analysis, Writing – review and editing. BT: Formal analysis, Writing – review and editing. PT: Conceptualization, Writing – review and editing. MS: Conceptualization, Writing – original draft, Writing – review and editing. GP: Data curation, Software, Writing – original draft, Writing – review and editing. FL: Formal analysis, Writing – review and editing. GA: Formal analysis, Writing – review and editing. SJL: Formal analysis, Writing – review and editing. RC: Conceptualization, Funding acquisition, Supervision, Validation, Writing – review and editing.

## References

[B1] Abarenkov K. Zirk A. Piirmann T. Pöhönen R. Ivanov F. Nilsson R. H. (2022). *UNITE QIIME Release for Fungi. Version 16.10.2022.* Irvine: UNITE Community. 10.15156/BIO/2483915

[B2] Barata A. Malfeito-Ferreira M. Loureiro V. (2012). The microbial ecology of wine grape berries. *Int. J. Food Microbiol*. 3 243–259. 10.1016/j.ijfoodmicro.2011.11.025 22189021

[B3] Belda I. Zarraonaindia I. Perisin M. Palacios A. Acedo A. (2017). Corrigendum: From vineyard soil to wine fermentation: Microbiome approximations to explain the “terroir” concept. *Front. Microbiol*. 8:1065. 10.3389/fmicb.2017.01065 28616012 PMC5467490

[B4] Bleidorn C. (2015). Third generation sequencing: technology and its potential impact on evolutionary biodiversity research. *Syst. Biodivers*. 14 1–8. 10.1080/14772000.2015.1099575

[B5] Bodor A. Bounedjoum N. Vincze G. E. Kis A. E. Laczi K. Bende G. (2020). Challenges of unculturable bacteria: environmental perspectives. *Rev. Environ. Sci. Biotechnol.* 19 1–22. 10.1007/s11157-020-09522-4

[B6] Bokulich N. A. Collins T. S. Masarweh C. Allen G. Heymann H. Ebeler S. E. (2016). Associations among wine grape microbiome, metabolome, and fermentation behavior suggest microbial contribution to regional wine characteristics. *mBio* 7:e00631-16. 10.1128/mbio.00631-16 27302757 PMC4959672

[B7] Bokulich N. A. Thorngate J. H. Richardson P. M. Mills D. A. (2014). Microbial biogeography of wine grapes is conditioned by cultivar, vintage, and climate. *Proc. Natl. Acad. Sci. U. S. A.* 111 E139–E148. 10.1073/pnas.1317377110 24277822 PMC3890796

[B8] Bougreau M. Ascencio K. Bugarel M. Nightingale K. Loneragan G. (2019). Yeast species isolated from Texas High Plains vineyards and dynamics during spontaneous fermentations of Tempranillo grapes. *PLoS One* 14:e0216246. 10.1371/journal.pone.0216246 31048913 PMC6497380

[B9] Bruez E. Vallance J. Gerbore J. Lecomte P. Da Costa J. P. Guerin-Dubrana L. (2014). Analyses of the temporal dynamics of fungal communities colonizing the healthy wood tissues of esca leaf-symptomatic and asymptomatic vines. *PLoS One* 9:e95928. 10.1371/journal.pone.0095928 24788412 PMC4006835

[B10] Callahan B. J. McMurdie P. J. Rosen M. J. Han A. W. Johnson A. J. Holmes S. P. (2016). DADA2: High-resolution sample inference from Illumina amplicon data. *Nat. Methods* 13 581–583. 10.1038/nmeth.3869 27214047 PMC4927377

[B11] Camilo S. Chandra M. Branco P. Malfeito-Ferreira M. (2022). Wine microbial consortium: Seasonal sources and vectors linking vineyard and winery environments. *Ferment.* 8:324. 10.3390/fermentation8070324

[B12] Caporaso J. G. Kuczynski J. Stombaugh J. Bittinger K. Bushman F. D. Costello E. K. (2010). QIIME allows analysis of high-throughput community sequencing data. *Nat. Methods* 7 335–336. 10.1038/nmeth.f.303 20383131 PMC3156573

[B13] Cocolin L. Alessandria V. Dolci P. Gorra R. Rantsiou K. (2013). Culture independent methods to assess the diversity and dynamics of microbiota during food fermentation. *Int. J. Food Microbiol.* 167 29–43. 10.1016/j.ijfoodmicro.2013.05.008 23791362

[B14] Dissanayake A. J. Purahong W. Wubet T. Hyde K. D. Zhang W. Xu H. (2018). Direct comparison of culture-dependent and culture-independent molecular approaches reveal the diversity of fungal endophytic communities in stems of grapevine (*Vitis vinifera*). *Fungal Divers.* 90 85–107. 10.1007/s13225-018-0399-3

[B15] Fernandes P. Afonso I. M. Pereira J. Rocha R. Rodrigues A. S. (2023). Epiphitic microbiome of Alvarinho wine grapes from different geographic regions in Portugal. *Biology* 12:146. 10.3390/biology12020146 36829425 PMC9952175

[B16] Gao F. Chen J. Xiao J. Cheng W. Zheng X. Wang B. (2019). Microbial community composition on grape surface controlled by geographical factors of different wine regions in Xinjiang, China. *Food Res. Int.* 122 348–360. 10.1016/j.foodres.2019.04.029 31229088

[B17] Gilbert J. A. van der Lelie D. Zarraonaindia I. (2014). Microbial terroir for wine grapes. *Proc. Natl. Acad. Sci. U. S. A.* 111 5–6. 10.1073/pnas.1320471110 24309378 PMC3890852

[B18] Grangeteau C. Roullier-Gall C. Rousseaux S. Gougeon R. D. Schmitt-Kopplin P. Alexandre H. (2017). Wine microbiology is driven by vineyard and winery anthropogenic factors. *Microb. Biotechnol*. 10 354–370. 10.1111/1751-7915.12428 27778455 PMC5328833

[B19] Iorizzo M. Sicilia A. Nicolosi E. Forino M. Picariello L. Lo Piero A. R. (2023). Investigating the impact of pedoclimatic conditions on the oenological performance of two red cultivars grown throughout southern Italy. *Front. Plant. Sci.* 14:1250208. 10.3389/fpls.2023.1250208 37780525 PMC10540683

[B20] Kamilari E. Mina M. Karallis C. Tsaltas D. (2021). Metataxonomic analysis of grape microbiota during wine fermentation reveals the distinction of Cyprus regional *terroirs*. *Front. Microbiol*. 12:726483. 10.3389/fmicb.2021.726483 34630353 PMC8494061

[B21] Kazou M. Pagiati L. Dotsika E. Proxenia N. Kotseridis Y. Tsakalidou E. (2023). The microbial terroir of the Nemea zone Agiorgitiko cv.: A first metataxonomic approach. *Aust. J. Grape Wine Res.* 2023:8791362. 10.1155/2023/8791362

[B22] Kecskeméti E. Berkelmann-Löhnertz B. Reineke A. (2016). Are epiphytic microbial communities in the carposphere of ripening grape clusters (*Vitis vinifera* L.) different between conventional, organic, and biodynamic grapes? *PLoS One* 11:e0160852. 10.1371/journal.pone.0160852 27500633 PMC4976965

[B23] Kioroglou D. Kraeva-Deloire E. Schmidtke L. M. Mas A. Portillo M. C. (2019). Geographical origin has a greater impact on grape berry fungal community than grape variety and maturation state. *Microorganisms* 7:669. 10.3390/microorganisms7120669 31835464 PMC6956300

[B24] Knight S. Klaere S. Fedrizzi B. Goddard M. R. (2015). Regional microbial signatures positively correlate with differential wine phenotypes: Evidence for a microbial aspect to terroir. *Sci. Rep*. 5:14233. 10.1038/srep14233 26400688 PMC4585847

[B25] Knight S. J. Karon O. Goddard M. R. (2020). Small scale fungal community differentiation in a vineyard system. *Food Microbiol.* 87:103358. 10.1016/j.fm.2019.103358 31948613

[B26] La Hens D. V. Bravo-Ferrada B. M. Delfederico L. Caballero A. C. Semorile L. C. (2014). Prevalence of *Lactobacillus plantarum* and *Oenococcus oeni* during spontaneous malolactic fermentation in Patagonian red wines revealed by polymerase chain reaction-denaturing gradient gel electrophoresis with two targeted genes. *Aust. J. Grape Wine Res.* 21 49–56. 10.1111/ajgw.12110

[B27] Li J. Hu W. Huang X. Xu Y. (2018). Investigation of yeast population diversity and dynamics in spontaneous fermentation of Vidal blanc icewine by traditional culture-dependent and high-throughput sequencing methods. *Food Res. Int.* 112 66–77. 10.1016/j.foodres.2018.06.011 30131160

[B28] Liu D. Howell K. (2021). Community succession of the grapevine fungal microbiome in the annual growth cycle. *Environ. Microbiol*. 23 1842–1857. 10.1111/1462-2920.1517 32686214

[B29] Lombardi S. J. Pannella G. Iorizzo M. Moreno-Arribas M. V. Tremonte P. Succi M. (2018). Sequential inoculum of Hanseniaspora guilliermondii and Saccharomyces cerevisiae for winemaking Campanino on an industrial scale. *World J. Microbiol. Biotechnol*. 34:161. 10.1007/s11274-018-2540-6 30357477

[B30] Lorenz D. H. Eichhorn K. W. Bleiholder H. Klose R. Meier U. Weber E. (1995). Growth stages of the grapevine: Phenological growth stages of the grapevine (*Vitis vinifera* L. ssp. vinifera)—Codes and descriptions according to the extended BBCH scale. *Aust. J. Grape Wine Res.* 1 100–103.

[B31] Martiniuk J. T. Hamilton J. Dodsworth T. Measday V. (2023). Grape-associated fungal community patterns persist from berry to wine on a fine geographical scale. *FEMS Yeast Res*. 23:foac067. 10.1093/femsyr/foac067 36592956 PMC9876423

[B32] Martins G. Vallance J. Mercier A. Albertin W. Stamatopoulos P. Rey P. (2014). Influence of the on the epiphytic yeasts and yeast-like fungi colonizing grape berries during the ripening process. *Int. J. Food Microbiol.* 177 21–28. 10.1016/j.ijfoodmicro.2014.02.002 24603471

[B33] McMurdie P. J. Holmes S. (2013). phyloseq: An R package for reproducible interactive analysis and graphics of microbiome census data. *PLoS One* 8:e61217. 10.1371/journal.pone.0061217 23630581 PMC3632530

[B34] Mezzasalma V. Sandionigi A. Bruni I. Bruno A. Lovicu G. Casiraghi M. (2017). Grape microbiome as a reliable and persistent signature of field origin and environmental conditions in Cannonau wine production. *PLoS One* 12:e0184615. 10.1371/journal.pone.0184615 28892512 PMC5593190

[B35] Morgan H. H. Du Toit M. Setati M. E. (2017). The grapevine and wine microbiome: Insights from high-throughput amplicon sequencing. *Front. Microbiol*. 8:820. 10.3389/fmicb.2017.00820 28553266 PMC5425579

[B36] Morrison-Whittle P. Goddard M. R. (2018). From vineyard to winery: A source map of microbial diversity driving wine fermentation. *Environ. Microbiol*. 20 75–84. 10.1111/1462-2920.13960 29052965

[B37] Morrison-Whittle P. Lee S. A. Goddard M. R. (2017). Fungal communities are differentially affected by conventional and biodynamic agricultural management approaches in vineyard ecosystems. *Agric. Ecosyst. Environ*. 246 306–313. 10.1016/j.agee.2017.05.022

[B38] Musetti R. Vecchione A. Stringher L. Borselli S. Zulini L. Marzani C. M. (2006). Inhibition of sporulation and ultrastructural alterations of grapevine downy mildew by the endophytic fungus *Alternaria alternata*. *Phytopathology* 96 689–698. 10.1094/PHYTO-96-0689 18943142

[B39] Pacifico D. Squartini A. Crucitti D. Barizza E. Lo Schiavo F. Muresu R. (2019). The role of the endophytic microbiome in the grapevine response to environmental triggers. *Front. Plant Sci.* 10:1256. 10.3389/fpls.2019.01256 31649712 PMC6794716

[B40] Papadopoulou E. Bekris F. Vasileiadis S. Papadopoulou K. K. Karpouzas D. G. (2022). Different factors are operative in shaping the epiphytic grapevine microbiome across different geographical scales: Biogeography, cultivar or vintage? *J. Sustain. Agric. Environ*. 1 287–301. 10.1002/sae2.12030

[B41] Patanita M. Albuquerque A. Campos M. D. Materatski P. Varanda C. M. Ribeiro J. A. (2022). Metagenomic assessment unravels fungal microbiota associated to grapevine trunk diseases. *Horticulture* 8:288. 10.3390/horticulturae8040288

[B42] Perpetuini G. Rossetti A. P. Battistelli N. Zulli C. Cichelli A. Arfelli G. (2022). Impact of vineyard management on grape fungal community and Montepulciano d’Abruzzo wine quality. *Food Res. Int.* 158:111577. 10.1016/j.foodres.2022.111577 35840262

[B43] Perpetuini G. Rossetti A. P. Giordano L. Pulcini M. Dufrusine B. Battistelli N. (2023). Characterization of Nero Antico di Pretalucente wine and grape fungal microbiota: An expression of Abruzzo region cultivar heritage. *Fermentation* 9:150. 10.3390/fermentation9020150

[B44] Pinto C. Pinho D. Sousa S. Pinheiro M. Egas C. Gomes A. C. (2014). Unravelling the diversity of grapevine microbiome. *PLoS One* 9:e85622. 10.1371/journal.pone.0085622 24454903 PMC3894198

[B45] Pretorius I. S. (2020). Tasting the terroir of wine yeast innovation. *FEMS Yeast Res*. 20:foz084. 10.1093/femsyr/foz084 31830254 PMC6964221

[B46] Rohart F. Gautier B. Singh A. Lê Cao K. A. (2017). mixOmics: An R package for ’omics feature selection and multiple data integration. *PLoS Comput. Biol*. 13:e1005752. 10.1371/journal.pcbi.1005752 29099853 PMC5687754

[B47] Rossetti A. P. Perpetuini G. Battistelli N. Zulli C. Arfelli G. Suzzi G. (2023). Capturing the fungal community associated with conventional and organic Trebbiano Abruzzese grapes and its influence on wine characteristics. *Food Biosci*. 52:102382. 10.1016/j.fbio.2023.102382

[B48] Salvetti E. Campanaro S. Campedelli I. Fracchetti F. Gobbi A. Tornielli G. B. (2016). Whole-metagenome-sequencing-based community profiles of *Vitis vinifera* L. cv. Corvina berries withered in two post-harvest conditions. *Front. Microbiol*. 7:937. 10.3389/fmicb.2016.00937 27445999 PMC4917526

[B49] Sanzani S. M. Sgaramella M. Mosca S. Solfrizzo M. Ippolito A. (2021). Control of *Penicillium expansum* by an epiphytic basidiomycetous yeast. *Horticuilture* 7:473. 10.3390/horticulturae7110473

[B50] Setati M. E. Jacobson D. Bauer F. F. (2015). Sequence-based analysis of the *Vitis vinifera* L. cv Cabernet Sauvignon grape must mycobiome in three south african vineyards employing distinct agronomic systems. *Front. Microbiol*. 6:1358. 10.3389/fmicb.2015.01358 26648930 PMC4663253

[B51] Sherman E. Coe M. Grose C. Martin D. Greenwood D. R. (2020). Metabolomics approach to assess the relative contributions of the volatile and non-volatile composition to expert quality Ratings of Pinot Noir wine quality. *J. Agric. Food Chem.* 68 13380–13396. 10.1021/acs.jafc.0c04095 32893630

[B52] Singh P. Gobbi A. Santoni S. Hansen L. H. This P. Péros J. P. (2018). Assessing the impact of plant genetic diversity in shaping the microbial community structure of *Vitis vinifera* phyllosphere in the Mediterranean. *Front. Life. Sci.* 11:35–46. 10.1080/21553769.2018.1552628

[B53] Takahashi M. Ohta T. Masaki K. Mizuno A. Goto-Yamamoto N. (2014). Evaluation of microbial diversity in sulfite-added and sulfite-free wine by culture-dependent and-independent methods. *J. Biosci. Bioeng*. 117 569–575. 10.1016/j.jbiosc.2013.10.012 24239025

[B54] Testa B. Lombardi S. J. Iorizzo M. Letizia F. Di Martino C. Di Renzo M. (2020). Use of strain *Hanseniaspora guilliermondii* BF1 for winemaking process of white grapes *Vitis vinifera* cv Fiano. *Eur. Food Res. Technol.* 246 549–561. 10.1007/s00217-019-03424-8

[B55] Testa B. Lombardi S. J. Tremonte P. Succi M. Tipaldi L. Pannella G. (2014). Biodiversity of *Lactobacillus plantarum* from traditional Italian wines. *World J. Microbiol. Biotechnol*. 30 2299–2305. 10.1007/s11274-014-1654-8 24817564 PMC4072923

[B56] Van Leeuwen C. Seguin G. (2006). The concept of terroir in viticulture. *J. Wine Res*. 17 1–10. 10.1080/09571260600633135

[B57] Varanda C. M. Oliveira M. Materatski P. Landum M. Clara M. I. Félix M. D. (2016). Fungal endophytic communities associated to the phyllosphere of grapevine cultivars under different types of management. *Fungal. Biol.* 120 1525–1536. 10.1016/j.funbio.2016.08.002 27890088

[B58] Verginer M. Leitner E. Berg G. (2010). Production of volatile metabolites by grape-associated microorganisms. *J. Agric. Food Chem*. 58 8344–8350. 10.1021/jf100393w 20575540

[B59] Vitulo N. Lemos W. J. F. Jr. Calgaro M. Confalone M. Felis G. E. Zapparoli G. (2019). Bark and grape microbiome of *Vitis vinifera*: Influence of geographic patterns and agronomic management on bacterial diversity. *Front. Microbiol*. 9:3203. 10.3389/FMICB.2018.03203 30671035 PMC6331396

[B60] Wei R. T. Chen N. Ding Y. T. Wang L. Gao F. F. Zhang L. (2022). Diversity and dynamics of epidermal microbes during grape development of Cabernet Sauvignon (*Vitis vinifera* L.) in the ecological viticulture model in Wuhai, China. *Front. Microbiol*. 13:935647. 10.3389/fmicb.2022.935647 35847061 PMC9280189

[B61] White T. J. Bruns T. Lee S. J. W. T. Taylor J. (1990). Amplification and direct sequencing of fungal ribosomal RNA genes for phylogenetics. *PCR Protocol* 18 315–322. 10.1016/B978-0-12-372180-8.50042-1

[B62] Xu X. Miao Y. Wang H. Du J. Wang C. Shi X. (2022). Analysis of microbial community diversity on the epidermis of wine grapes in Manasi’s vineyard, Xinjiang. *Foods* 11:3174. 10.3390/foods11203174 37430923 PMC9602134

[B63] Zarraonaindia I. Owens S. M. Weisenhorn P. West K. Hampton-Marcell J. Lax S. (2015). The soil microbiome influences grapevine-associated microbiota. *mBio* 6:e02527-14. 10.1128/mbio.02527-14 25805735 PMC4453523

[B64] Zhang J. Zhang B. Liu Y. Guo Y. Shi P. Wei G. (2018). Distinct large-scale biogeographic patterns of fungal communities in bulk soil and soybean rhizosphere in China. *Sci. Total. Environ*. 664 791–800. 10.1016/j.scitotenv.2018.07.016 29990927

