# Supplementary figures and images for: Diversity of fungal communities on Cabernet and Aglianico grapes from vineyards located in Southern Italy
Source: Front Microbiol. 2024 Apr 25;15:1399968. doi: 10.3389/fmicb.2024.1399968 (PMC11079197; doi:10.3389/fmicb.2024.1399968)

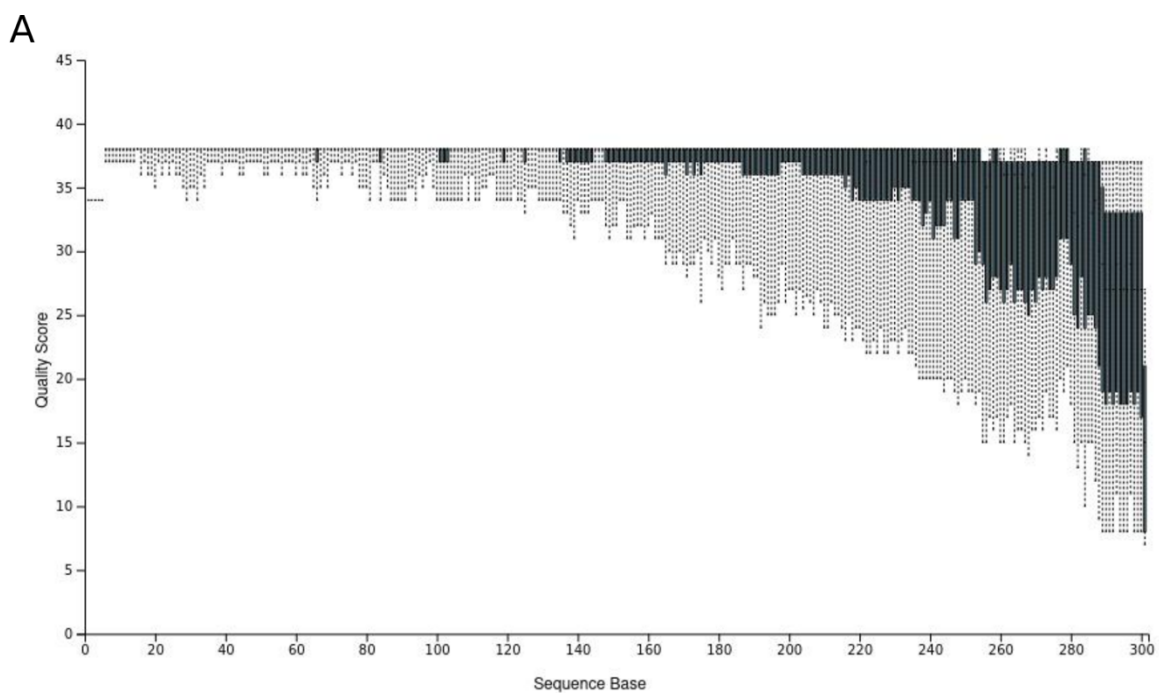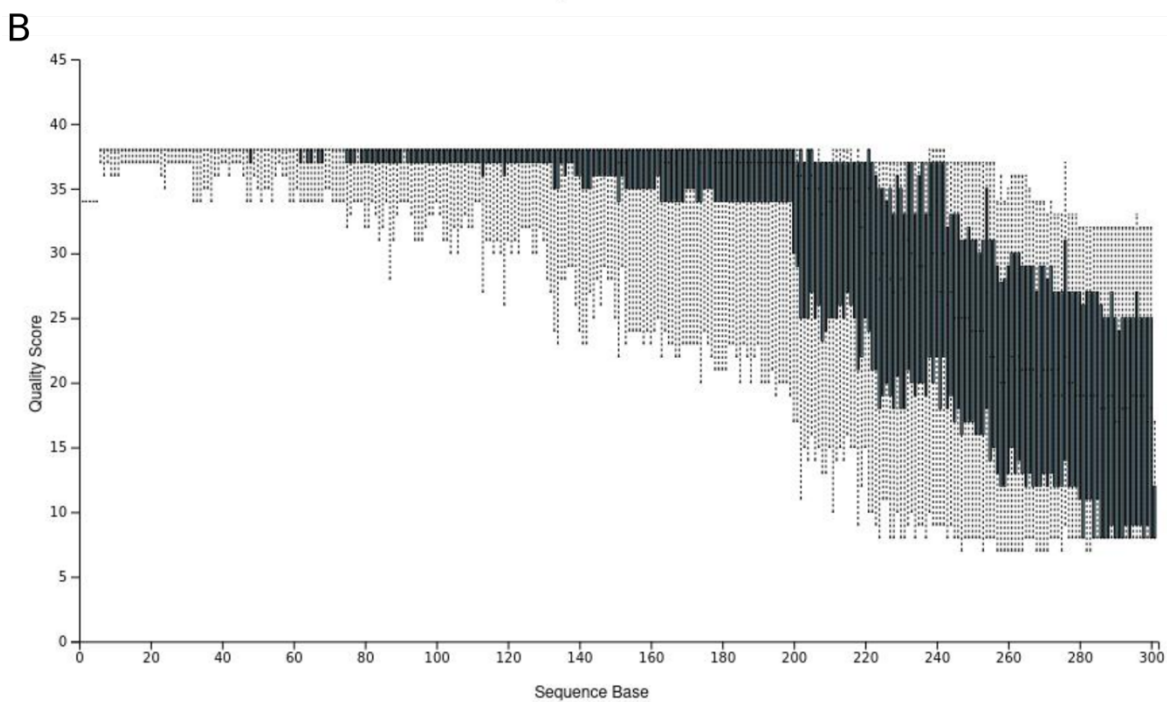

Supplementary Figure 1. Quality score plot of forward reads (A) and reverse reads (B).

Supplement: Supplementary file 1 [file Image_1.pdf]
