# Supplementary material for: Diversity of fungal communities on Cabernet and Aglianico grapes from vineyards located in Southern Italy
Source: Front Microbiol. 2024 Apr 25;15:1399968. doi: 10.3389/fmicb.2024.1399968 (PMC11079197; doi:10.3389/fmicb.2024.1399968)
Supplement: Supplementary file 2 [file Image_2.pdf]

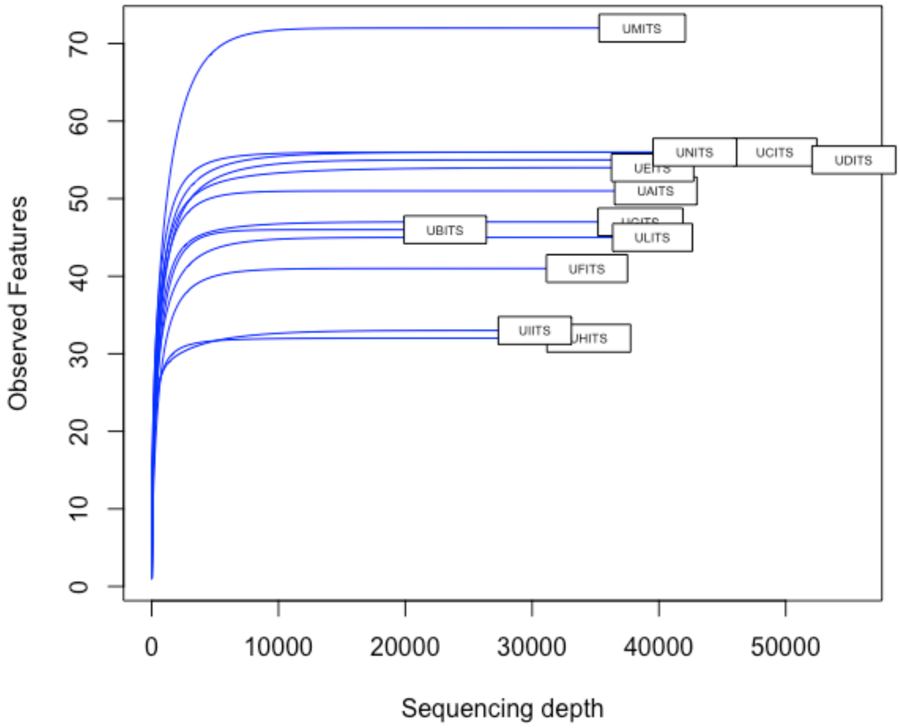

Supplementary Figure 2. Rarefaction curves showing the number of observed features in relation to the number of sequenced reads (Sequencing depth).
